# Workplace Demands and Cognitive Health Inequities Across Race and Ethnicity: A Scoping Review

**DOI:** 10.1093/geroni/igab046.084

**Published:** 2021-12-17

**Authors:** Ernest Gonzales, Rachel Krutchen, Cliff Whetung, Jane Lee

**Affiliations:** 1 New York University, New York, New York, United States; 2 NYU, New York, New York, United States; 3 University of Hawai'i, Myron B. Thompson School of Social Work, Honolulu, Hawaii, United States

## Abstract

This PRISMA informed scoping review sought to understand the longitudinal association between workplace demands with cognitive health; and to review how race and ethnicity are investigated in this area of research and evidence of moderating effects. Peer-reviewed articles were drawn from five databases. Inclusion criteria were populations aged 18+, broad conceptualization of workplace demands (e.g., occupational complexity, mental work demands), and cognitive health outcomes (e.g., cognitive functioning, ADRD). The majority of studies drew from theories that did not interrogate heterogeneity and diverse aging experiences. Consequently, the majority of studies (85%) did not investigate inequities by race and ethnicity although variables and methods are available. Cognitive health inequities are evidenced but findings are mixed and more rigorous causal research is needed. We discuss integrating emerging critical theories (e.g. Critical Race Theory, critical gerontology, minority stress) to sharpen the focus on racial health inequities in an emerging area of prevention research.

